# Trauma Informed Interventions to Reduce Seclusion, Restraint and Restrictive Practices Amongst Staff Caring for Children and Adolescents with Challenging Behaviours: A Systematic Review

**DOI:** 10.1007/s40653-023-00524-2

**Published:** 2023-03-15

**Authors:** Peter Kelly, Mohamad M. Saab, Emma J. Hurley, Sinéad Heffernan, John Goodwin, Zamzaliza A. Mulud, Maria O Malley, James O Mahony, Margaret Curtin, Gunter Groen, Svetla Ivanova, Astrid Jörns-Presentati, Joonas Korhonen, Kostadin Kostadinov, Mari Lahti, Valentina Lalova, Gergana Petrova, Aine O Donovan

**Affiliations:** 1 School of Nursing and Midwifery, Trinity College Dublin, Dublin, Ireland; 2 School of Nursing and Midwifery, University College Cork, Cork, Ireland; 3 Centre for Nursing Studies, Universiti Teknologi MARA Selangor, Puncak Alam, Selangor, Malaysia; 4 Sciences Hamburg Department of Social Work, University of Applied, Hamburg, Germany; 5 Department of Nursing Care, Faculty of Public Health, Medical University of Plovdiv, Plovdiv, Bulgaria; 6 Department of Social Work, University of Applied Sciences, Hamburg, Germany; 7 Faculty of Health and Well-Being, Turku University of Applied Science, Turku, Finland; 8 Department of Social Medicine and Public Health, Faculty of Public Health, Medical University of Plovdiv, Plovdiv, Bulgaria

**Keywords:** Trauma informed care, Children, Adolescents, Coercive practice, Interventions, Violence and aggression

## Abstract

Engaging with children and adolescents in mental health settings who are exhibiting behaviours that challenge can often result in the use of seclusion, restraint and coercive practices. It is recognised that more therapeutic ways to engage this population are needed, adopting trauma informed interventions may provide a solution. The aim of this systematic review is to synthesize the evidence in relation to the effect of trauma-informed interventions on coercive practices in child and adolescent residential settings. The review is guided by elements of the Cochrane Handbook for Systematic Reviews of Interventions and reported using the Preferred Reporting Items for Systematic reviews and Meta-Analyses (PRISMA) checklist. Results were synthesized and reported narratively. Nine studies met the eligibility criteria for this review. There was a lack of homogeneity amongst the studies. The trauma-informed interventions used were typically multi-faceted, underpinned by a variety of approaches and sought to bring about changes to clinical practice*.* Most studies (n = 8) reported significant reductions in the use of restrictive practices following the implementation of a trauma informed approach. The use of a trauma-informed approach, underpinned by an organisational change or implementation strategy, have the potential to reduce coercive practices with children and adolescents. However, the included interventions were insufficiently described to draw strong conclusions.

## Introduction

The “United Nations Convention on the Rights of the Child” (UNCRC), ratified by almost all nations of the world, states the fundamental rights of children and especially the right to life, health, and development, bans discrimination, mandates the protection of children’s interests (Save the Children, 2022). Children and young people who require physical and mental health care in healthcare settings can at times present with behaviour that can challenge care provision and responses to this occupy a ‘contested space’. Traditionally, challenging or aggressive behaviour was managed using practices that included the coercive use of chemical and sometimes physical restraints (National Institute for Health and Care Excellence, 2017). However, coercive practices such as the seclusion, restraint and the use of time out for managing behaviours that challenge in young people have been linked with negative psychological consequences for young mental health service users (De Hert et al., 2011; LeBel et al., 2010).

Utilising coercive practices can traumatise and/or retraumatise a young person who may have experienced adversity in life previously, as many safety procedures designed to reduce unsafe behaviour can trigger a young person who has experienced trauma and can induce dysregulated states (Hodgdon et al., 2013). This in turn can escalate rather than deescalate the behaviour, creating emotional and physical safety risks. Furthermore, evidence suggests that coercive practices can cause service users to feel frightened (Steckley & Kendrick, 2008) and to experience hyper vigilance (Brophy et al., 2016) and distress when peers are restrained (Snyder & Kendrick, 2018). This can inadvertently damage the therapeutic relationship with healthcare staff (SAHMSA, 2014; Steckley & Kendrick, 2008). Coercive practices can also negatively impact staff who care for service users who have experienced trauma. This can result in staff experiencing secondary trauma or burnout (Beattie et al., 2019), consequently reducing staff capacity to provide therapeutic care and resulting in negative health outcomes for the staff member (Bloom, 2013). Coercive practices are also linked with organisational issues such as staff retention (Craig & Sanders, 2018), decreased length of stay, service user and staff injuries, and workers’ compensation claims (Forrest et al., 2018).

As a result, services are recognising the need to find ways to engage with children and adolescents in more therapeutic ways. One such approach that underpins frameworks to reduce coercive practices is Trauma Informed Care (TIC). This approach suggests that behind all behaviours that challenge is an unmet need (SAHMSA, 2014) and strongly promotes developing therapeutic engagement approaches that can enhance communication and explore the young person’s needs. Evidence suggests that patient-centred interventions that use a trauma informed approach to enhance de-escalation can result in reduced coercive practices (Griffing et al., 2021; Matte-Landry & Collin-Vézina, 2022). Utilising a trauma informed approach can also equip staff to deliver better patient care (Elwyn et al., 2015; Griffing et al., 2021) through focusing on staff’s capacity to be therapeutic and seeking to improve job satisfaction (Hidalgo et al., 2016).

TIC is characterised by a strength-based approach (Forrest et al., 2018) to therapeutically engage with children and adolescents to reduce coercive practices, using both staff and service user focused approaches. These include psychoeducational training designed to build staff effectiveness (Griffing et al., 2021), service-user focused interventions including play-based (Hidalgo et al., 2016), and sport-based interventions (D’Andrea et al., 2013), as well as debriefing and problem-solving approaches (Azeem et al., 2015). The development of a trauma-informed milieu (Brown et al., 2013) that utilises sensory-based alternatives to reduce coercive practices has also been suggested (Denison et al., 2018).

Evidence suggests that TIC-based strategies have positively impacted the mental health of service users including reduced rates of post-traumatic stress disorder (PTSD) symptoms reported reduced externalizing and internalizing behaviours (Hodgdon et al., 2013; Marrow et al., 2012) and increases in service users' feelings of safety (Elwyn et al., 2015). Furthermore, greater improvements in functional impairment, and reduced length of admission (Boel-Studt, 2017) are reported. Some evidence suggests that changes made have been sustained in practice and further developed (Hale & Wendler, 2020; Matte-Landry & Collin-Vézina, 2022). While there is an increasing body of literature on TIC, to the best of the authors’ knowledge, no prior review has systematically reported on trauma-informed interventions as they relate to caring for children and adolescents in both mental health and paediatric settings. The purpose of this systematic review was to synthesize evidence in relation to using trauma-informed interventions to reduce coercive practices in child and adolescent residential settings.

## Methods

This systematic review is reported using the Preferred Reporting Items for Systematic reviews and Meta-Analyses (PRISMA) checklist (Page et al., 2021) and is guided by elements of the Cochrane Handbook for Systematic Reviews of Interventions (Higgins et al., 2019).

### Inclusion and Exclusion Criteria

The review eligibility criteria were pre-determined according to the review aims and were formulated using the modified Population, Intervention, Comparison, and Outcome (PICO) framework (Schardt et al., 2007), to include “S” for Study design and “S” for Setting (i.e., PICOSS). The inclusion criteria were: Population: Any member of staff caring for children and adolescents (≤ 19 years); Intervention: Any trauma-informed intervention aimed to reduce seclusion, restraint, and coercive practices among staff; Comparison: Studies with/without comparators; Outcomes: Restrictive practices such as seclusion, use of restraints, and coercion used by staff (primary outcome) and/or any staff/patients/service user outcomes focused on non-restrictive practices (secondary outcome); Study design: Any primary research (including qualitative, quantitative descriptive, randomised controlled trials, non-randomised controlled trials, any pre-post designs); Setting: any child and adolescent residential setting. Studies with staff caring for adults (> 19 years) and interventions not aimed at reducing seclusion, restraint, and coercive practices were excluded. Editorials, opinion pieces, theses, dissertations, literature reviews, and conference abstracts were also excluded.

### Search Strategy

Electronic databases Academic Search Complete, MEDLINE, CINAHL, APA PsycArticles, APA PsycInfo, SocINDEX, and ERIC were searched in November 2021. The following keywords were truncated “*” to maximise retrieval, searched based on title and abstract, and combined used Boolean operators “OR” and “AND” as follows: (Child* OR adolescen* OR teen* OR kid OR kids OR paediatric* OR pediatric* OR "young person*" OR "young people*" OR youth*) AND (Seclu* OR restrain* OR coerc* OR isolat* OR de-escalat* OR deescalat* OR de-stimulat* OR destimulat* OR diffus* OR calm* OR "non aversive" OR non-aversive OR "non confront*" OR non-confront* OR constrain* OR lock* OR padded OR time-out OR "time out" OR timeout) AND (Trauma-inform* OR "trauma inform*" OR trauma-focus* OR "trauma focus*" OR trauma-based OR "trauma based" OR trauma-sensitive OR "trauma sensitive" OR trauma-aware* or "trauma aware*" OR safeguard*). The search was limited to studies published in English. No other limiters were used in order to maximise retrieval.

### Data Extraction and Synthesis

Records were screened in Covidence, an online software used to produce and manage systematic reviews (The Cochrane Collaboration, 2022). First, titles and abstracts were screened, and irrelevant papers were excluded. The full texts of potentially eligible papers were then obtained and screened. Each paper had to be screened twice by two independent reviewers **[PK]** and **[EH]**. A third independent reviewer **[MS]** resolved screening conflicts. Data from the included studies were extracted using a standardised data extraction table (Kelly et al., 2017; Saab et al., 2021), under the following headings: Author; country; aim; design; theoretical underpinning; sample; setting; instruments; intervention; implementation strategy; relevant outcomes measured; results; and further comments/other key findings. Due to the heterogeneity in study design, outcomes, and instruments, a meta-analysis was not possible. Therefore, results from the included studies were synthesised narratively (Lisy & Porritt, 2016).

### Quality Appraisal

The methodological quality of the included studies was appraised using the Mixed Methods Appraisal Tool (MMAT) (Hong et al., 2018). The MMAT assists in assessing the quality of five study categories: qualitative studies, randomised controlled trials (RCTs), non-randomised studies, quantitative descriptive studies, and mixed methods studies. In the current review the quality of three study categories were assessed, quantitative non-randomised (seven quality appraisal items), quantitative descriptive (twelve quality appraisal items), mixed methods (seventeen quality appraisal items) and qualitative studies (seven quality appraisal items). Each study was appraised by one researcher **[MOM]** and cross checked by three researchers **[ZAM,JOM,MC]**. Each appraisal item was voted on a “Yes”, “No”, and “Can’t tell” basis. Conflicts in quality appraisal were discussed until consensus was reached.

## Results

### Study Selection

**Fig. 1:**
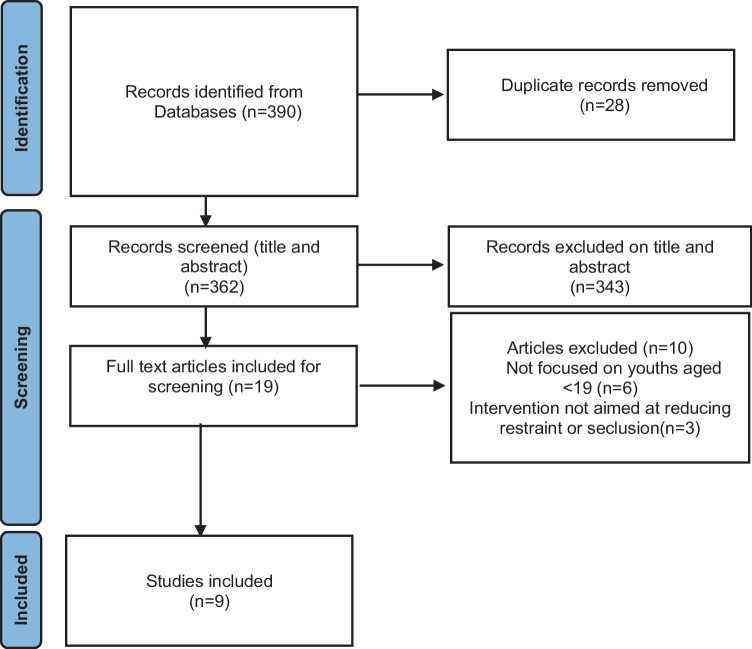
PRISMA flowchart

A total of 390 records were identified through database searching. Following deletion of duplicates, 362 records were screened based on title and abstract and 343 irrelevant records were excluded. The full text of the remaining 19 records was screened. Of those, nine were included in the current review. The PRISMA flow chart is available in Fig. 1.

### Quality Appraisal

All nine articles had clear aims, adequately addressed with the data collected. All studies (n = 9) reported that participants were representative of the target population and appeared to administer the intervention as intended. Measurements were appropriate in relation to all studies bar one (Denison et al., 2018), which was undefinable. Only one study Boel-Studt (2017) seemed to account for confounding variables in design or analysis whilst most studies (n = 6) provided complete outcome data. Both mixed methods and qualitative studies (Caldwell et al., 2014; Hidalgo et al., 2016) provided integrated components to answer the research questions and interpret findings. The full quality appraisal results are presented in Table 1.

**Table 1:** Quality Appraisal

**Study designs**	**Author(s) & year**	Quality appraisal items*																
		**1**	**2**	**3**	**4**	**5**	**6**	**7**	**8**	**9**	**10**	**11**	**12**	**13**	**14**	**15**	**16**	**17**
Quantitative non-randomised studies**	Boel-Studt (2017)	Y	Y	Y	Y	Y	Y	Y										
	Denison et al. (2018)	Y	Y	Y	CT	N	CT	Y										
	Marrow et al. (2012)	Y	Y	Y	Y	Y	N	Y										
Quantitative Descriptive***	Azeem et al. (2017)	Y	Y	Y	Y	N	CT	Y	Y	Y	Y	Y	N					
	Azeem et al. (2015)	Y	Y	Y	Y	Y	CT	Y	Y	Y	Y	Y	Y					
	Brown et al. (2013)	Y	Y	Y	Y	Y	CT	Y	Y	Y	Y	Y	Y					
	Hale and Wendle (2020)	Y	Y	Y	Y	N	N	Y	CT	CT	Y	CT	CT					
Mixed methods****	Hidalgo et al. (2016)	Y	Y	Y	Y	Y	CT	Y	Y	Y	Y	Y	Y	Y	Y	Y	Y	Y
Qualitative Case-Study *****	Caldwell et al., 2014	Y	Y											Y	Y	Y	Y	Y

*CT* can’t tell, *N* no, *Y* yes

^*^All studies: 1 = Clear research questions/aims; ^**^Non-randomised studies: 3 = Participants representative of target population 4 = Measurements appropriate regarding both the outcome and the intervention 5 = Complete outcome data 6 = Confounders accounted for in the design and analysis 7 = The intervention administered as intended; ^***^ Quantitative Descriptive 8 = Sampling strategy relevant to address the research question 9 = Sample representative of the target population 10 = The measurements are appropriate 11 = Risk of nonresponse bias is low 12 = Statistical analysis appropriate to answer the research question; ^*****^ Mixed methods 13 = Adequate rationale for using a mixed-methods design 14 = Components of the study effectively integrated to answer the research question 15 = Outputs of the integration of qualitative and quantitative components adequately interpreted 16 = Divergences and inconsistencies between quantitative and qualitative results adequately addressed 17 = Components of the study adhere to the quality criteria of each tradition of the methods involved

### Study Characteristics

A comprehensive overview of the included studies is provided in Table 2. The included studies reported using a range of methodological approaches, including: reports or case studies on trauma informed service initiatives (n = 5), quasi-experimental studies (n = 2), a retrospective study (n = 1) and a mixed methods study (n = 1). All studies were conducted in the United States of America and were underpinned by a range of trauma informed approaches. A range of settings were described in the included papers; the majority of studies incorporated either low or high security in-patient psychiatric treatment services, but two papers reported on studies conducted in correctional facilities (Marrow et al., 2012) and secure centres for child immigrants (Hidalgo et al., 2016). All the included studies were conducted in more than one service unit or ward. Only five of the included studies provided a sample size which ranged between 62 (Denison et al., 2018) and 458 (Azeem et al., 2017) participants. Seven of the included studies focused on an outcome of reduced use of restrictive practices for addressing challenging behaviour. One paper, aquasi-experimental study by Denison et al. (2018), focused on the impact of a training programme on staff attitudes towards seclusion and restraint.

**Table 2:** Extraction Table

**1. Author** **2. Country**	**3. Aim** **4. Design** **5. Theoretical underpinning**	**6. Sample** **7. Setting**	**8. Instruments**	**9. Intervention** **10. Implementation Strategy**	**11. Relevant Outcomes Measured**	**12. Results** **13. Further Comments/Other Key Findings**
1. Azeem et al. (2017) 2. United States	3. To determine effectiveness of 6 core strategies based on Trauma Informed Care (TIC) in reducing seclusion and restraint 4. Retrospective study 5. TIC	6. Children and Adolescent (n = 458) in-patients 7. Child and adolescent in-patient hospital with 26 beds: 3 units; 9 bed (f) unit, 9 bed (m) for adolescents & an 8 bed unit (m + f) for younger children	8. Audit tool developed by investigators; demographics, admissions and incidence of seclusion and restraint	9. Administrative and frontline staff received training in TIC; The Six Core Strategies© (Huckshorn et al., 2005) leadership toward organizational change, use of data to inform practice, workforce development, use of restraint and seclusion reduction tools, improve consumer’s role, and debriefing techniques. 10. Six-core strategies contains an implementation component	11. (i) Reductions in restraints (ii) Reductions in seclusions	12. During six months pre-training there were 93 episodes (73 seclusions/20 restraints) involving 22 children and adolescents (f = 11/m = 11). In the six months post-training there was 31 episodes (6 seclusions/25 restraints) involving 11 children or adolescents (f = 7/m = 4) 13. Major diagnosis of those placed in seclusion or restraint was behaviour disorder (61%) and mood disorder (52%)
1. Azeem et al. (2015) 2. United States	3. To reduce the use of restraints and to provide trauma informed care in a 52-bed Paediatric Psychiatric Hospital 4. Case Report 5. Primary prevention principles based on trauma-informed and strength-based care	6. N/A 7. 52-bed Paediatric Psychiatric Hospital USA	8. Measurement of frequency of incidents over 10 years	9. Trauma informed training, The Six Core Strategies© (Huckshorn et al., 2005) leadership Staff involvement and training community. Debriefing and proble- solving Involvement of youth and family in treatment planning Training in de-escalation techniques. 10. Use of frequency data to motivate change	11. Incidents of mechanical restraints, incidents of physical restraints	12. Over a 10-year period, mechanical restraints decreased by 100%, from 485 in 2005 to “zero” in 2014 and none in the last 3 years. Physical restraints decreased by 88%, from 3,033 in 2005 to 379 in 2014. Mechanical N = 485 at yr 1 to 0 in yr 10Physical N = 3033 at y1 to 379 at yr 10 13. Individualised treatment plans updated to reflect training principles and buy-in and participation from patients, families, and staff
1. Boel-Studt (2017) 2. United States	3. To examine the effectiveness of a TI Psychiatric Residential Treatment Approach (TI-PRT) versus a traditional Psychiatric Residential Treatment (PRT) approach 4. Quasi-experimental study 5. TIC	6. Trauma-affected children & adolescents (n = 205) in receipt of PRT (n = 100) or TI-PRT (n = 105) 7. Various psychiatric residential treatment settings	8. Child and Adolescent Functional Assessment Scale (CFAS) (Hodges et al., 2004)	9. Intervention models: (i) Trauma Informed Psychiatric Residential Treatment (TI-PRT) (ii) Traditional Psychiatric Residential Treatment (TRT) 10. Fidelity to TI-PRT model was assessed using a training and implementation check-list, created for the project	11. (i) Number of physical restraints and seclusions (ii) Reductions in functional impairment (iii) length of time in treatment (iv) uptake of community-based placements *Influence of co-variates was also considered e.g.; age, race, gender and trauma histories	12. Incidents of seclusion for youth in the TI-PRT programme was less when compared with PRT [p < 0.001]. Incidents of restraint were reported as being higher in the TI-PRT group [p < 0.01] 13. The TI-PRT group experienced greater decreases in functional impairment over time [p = 0.000]. Receiving TI-PRT v’s PRT accounted for 25% of variance of time spent in treatment. Being older was associated with increased restraint [p = 0.002] and less seclusion [p = 0.014] for both groups. Higher levels of functioning were associated with less seclusion [p = 0.000]. Those discharged from TI-PRT v’s PRT were reported to have had higher levels of functioning.
1. Brown et al. (2013) 2. United States	3. To describe the adaption and implementation of a systems orientated Trauma Systems Therapy [TST] model (Saxe et al., 2007) at 3 residential care facilities for young people 4. 3-site Case Study 5. Trauma Systems Therapy	6. Residential Treatment Settings [n = 3] 7. Residential Treatment Centre [RTC] for (i) children with mental health issues (ii) immigrant children who have committed petty crime (iii) Integrated child welfare and mental health provider	8. n/a	9. Trauma Systems Therapy [TST] model (Saxe et al., 2007) 10. Mixed implementation strategies across centres	11. In centre (i) reductions in seclusion and restraint (ii) Emotional regulation Stability and stability of the social environment (iii) Measured pre and post implementation of the TST programme- using Child and Adolescent Functional Assessment Scale [CAFAS] (Hodges, 1999) to measure eight domains of functioning	12. It was reported that following the implementation of the TST programme; centre (i) reduced seclusion, restraint and injury episodes from 80 to under 10 in the first 7 months, & centre (iii) over an 11-month period reduced in rates of ‘safety holds’ [restraint] per 1000- patient days from a peak of 42.98 to 7.59 13. It was reported that centre (ii) noted an improvement in emotional and environmental stability in one subject examined in a separate case study. It was reported that centre (iii) observed significant improvements in CAFAS scores when pre and post TST introduction was compared
1. Caldwell et al. (2014) 2. United States	3. To describe experiences of child and family serving programmes [n = 3] in implementing initiatives to reduce seclusion and restraint 4. Report on a 3-site case study 5. TIC- Six Core Strategies ©	6. [n = 3] child and family serving programmes 7. (i) Children’s mental health in-patient unit with 52 beds (ii) A youth development institute which provides a mix of in-patient and out-patient programmes for children 10-18yrs (iii) A residential programme that serves 101 males aged 11 to 21* *outside inclusion criteria	8. n/a	9. The Six Core Strategies© (Huckshorn et al., 2005) 10. Guided using the Six Core Strategies ©	11. In centre’s (i) use of mechanical restraint, use of physical restraint and seclusions (ii) number of restraints per month	12. In centres; (i) it was reported that from 2005 to 2013 the use of mechanical restraints reduced by 100%, the use of physical restraints reduced by 87%, and the use of seclusion reduced by 67% (ii) Over a 2-year period, restraints per month reduced from a peak of 49 to 0. 13. Centre (iii) was outside the inclusion criteria but reported a reduction in restraints by 75% over a 2-year period following the introduction of the Six Core Strategies ©
1. Denison et al. (2018) 2. United States	3. (i) To explore residential treatment staff’s attitudes towards seclusion and restraint (ii) To examine the impact of training on TIC and Ayers Sensory integration principles on staff attitudes to seclusion and restraint 4. Quasi-experimental study 5. TIC. Adult learning theory, TIC and SIP underpinned the educational intervention	6. Frontline Health Care Practitioners: pre training sample [n = 62] of which [n = 22] completed a post training questionnaire 7. Residential treatment centre for severe emotional and behavioural problems with approx. 60 residents [m] + [f]	8. Survey instrument was developed from; (i) ‘The Professionals Attitudes Towards Seclusion’ (Van Doeslaar et al., 2008) and (ii) the ‘Questionnaire for Organizational Assessment’ (Colton & Xiong, 2010)	9. A tailor-made in service training programme lasting 1 h- underpinned by Ayers Sensory Integration Principles (Ayers, 1972) and TIC principles 10. Adult learning theory (Knowles, 1984)	11. Staff attitudes towards: (i) TIC principles (ii) avoidance of restraint (iii) sensory integration process	12. Six statements; one on TIC [p = 0.049], one on alternatives for restraint [p = 0.047], and 4 on SIR [p = 0.031, p = 0.015, p = 0.036, p = 0.010] demonstrated statistically significant improvements in attitudes following training. Position, education levels experience and age were reported as being associated with better attitudes to TIC, restraint, and SIR following training [no p-values provided]. 13. Not statistically significant; Staff over 40yrs tended to agree with TIC principles more [p = 0.082]. Staff members with 4yrs + experience in an RTC tended to agree more with statements which supported the avoidance of seclusion. Staff with a college degree tended to support statements supporting SIR more [no p values provided for these results]
1. Hidalgo et al. (2016) 2. United States	3. To measure impact of a play-based, trauma-informed training program (PATHS to resilience) for staff working in shelters for unaccompanied migrant youth on (i) the quality of relationships amongst staff (ii) indicators of vicarious trauma as measured by changes in beliefs about safety, trust, intimacy, self-esteem and control (iii) job satisfaction (iv) job performance, residential facility frequency of restraints, medication use and critical behavioural incident**s** (v) staff beliefs regarding the residential facilities capacity to address mental health issues. 4. Mixed methods Pre-post measures taken pre, 6 months and 12 months post training. Qualitative interviews 5. TIC	6. 297 **r**esidential care staff in four pilot centres 7. Shelters for unaccompanied migrant youth (three low-risk, one high security)	8. **T**he Trauma Attachment Belief Scale (TABS) (Pearlman, 2003), the Mental Health Capacity Instrument (MHCI) (Feigenberg et al., 2010). The Andrews and Withey Job Satisfaction Questionnaire (Andrews and Withey, 1976).	9. PATHS to Resilience (PATHS) based on TST (Saxe et al., 2007) and Playfulness Training (Life is good Inc. 2012). An integration of play-based training and trauma-informed care (trauma systems therapy). Designed to reduce staff burnout and client negative behaviours. 10. Training was delivered to staff in a workshop style manner.	11. Administrative records 6–12 months post training were reviewed to ascertain; Incidents of restraints, frequency of the use of psychotropic medications, mental health interventions, aggression, and behaviourally-related critical incidents. Qualitative interviews (n = 15) explored broader implications of implementation of PATHS to resilience programme.	12. Residential facilities reported a decrease in number of restraints from baseline to 12 months (p = 0.359) after participating in the PATHS training. Content analysis of qualitative interviews show a positive orientation to the training and outcomes, in particular less restraint. 13. Reductions were also seen with regard to the use of psychotropic medications (p = 0.603), mental health interventions (p = 0.755), and behaviourally related critical incidents (p = 0.125). At the 12-month assessment, staff reported statistically significant decreases in distress from baseline on Self Safety, Other Safety, Other Trust, Other Esteem, Self-Intimacy, Other Intimacy, Self-Control, and the TABS total score. Staff perceived their residential facility to have a significantly higher level of mental health capacity at 12 months post-training. There was statistically significant improvement on TABS scale for youth care workers.
1. Marrow et al. (2012). 2. United States	3. To report on the evaluation of the efficacy of a Trauma informed program which focused on (i) reductions in post traumatic stress symptoms (ii) threats towards staff (iii) seclusion and restraint rates 4. A nonrandomized program evaluation study of a trauma-focused intervention for youth incarcerated for felony-level offenses in a juvenile justice setting 5. TIC	6. Youth (n = 74) aged 11–19 in two groups (i) Treatment as usual [TAU] (n = 38) & (ii) Trauma informed intervention (n = 36). Groups were allocated on the basis of unit protocol. 7. Two Units (i) TAU (ii) TIC informed Intervention unit, within a moderate-high security correctional facility mental health facility.	8. TARGET; a 10-session manualized treatment and prevention intervention for traumatized adolescents and adults (Ford & Russo, 2006). The Mood and Feelings Questionnaire (MFQ) (Angold et al., 1987). The Self-Report for Childhood Anxiety Related Disorders (SCARED; Birmaher et al., 1995). The Trauma Events Screening Inventory (Ford & Rogers, 1997). The UCLA PTSD Reaction Index. The Ohio Scales (OS; Ogles et al., 2001). The Generalized Expectancies for Negative Mood Regulation (NMR; Catanzaro & Mearns, 1990) The Massachusetts Youth Screening Instrument (MAYSI2) (Grisso & Barnum, 1998)	9. Three components (i) a one-day psychoeducational general trauma training on childhood traumatic stress. (ii) two-day training on Trauma Affect Regulation: Guide for Education and Therapy (TARGET) principles Three months supervision. (iii) Modifications to the unit environments with a goal of reducing trauma triggers. 10. TIC TARGET	11. Comparison of rates of seclusion and restraint between TAU and intervention groups between T1 and T2 and follow up. Incident reports were used to measure frequency.	12. At T1 Both groups used physical response (restraint) at the same rate. Measures at T2 showed that the TAU group used physical response (restraint) at a rate five times that of the intervention group. A similar trend emerged with use of seclusion and the number of menacing threats made by youth. Over time the TAU group used seclusion at a rate six times that of the intervention group. Additionally, the intervention group evidenced a continued reduction in the use of seclusion for eight months following the introduction of the intervention. 13. There was a similar pattern in the use of physical response and threats by youth over this time (declining in the treatment group).
1. Hale & Wendler (2020) 2. United States	3. To report on an evidence-based practice-informed implementation of trauma-informed care (TIC; SAHMSA, 2014). Aimed at reducing incidents of physical restraint and seclusion. 4. Quality improvement project. 5. TIC & IOWA model of Evidence based practice-revised	6. Children aged 4–11 yrs and adolescent ages 12–17 (n = not specified) 7. 97 bed Inpatient psychiatric hospital	8. N/A	9. Grounded within TIC and based on the Six Core Strategies © 10. Iowa Model for Evidence Based Practice & Revised, six core strategies. TIC, developed by NASMHPD (Azeem et al., 2011)	11. Frequency of physical restraint and seclusion- as indicated by incident reports	12. Data obtained at 6 months revealed a 41% reduction in the use of holds and seclusions, and a further 9% at 12 months post intervention 13. This hospital’s rates of use of seclusions and physical holds were consistently above expected benchmarks (75% above). Restraint frequently preceded seclusion prior to TLC implementation – changes were made to this practice. Patient’s care plans retooled to reflect TIC principles (data driven actions). Number of clients in the facility at any one time is not clear.

### Synthesis of Results

The trauma-informed interventions used in the included studies were typically multi-faceted in that they sought to bring about changes to *clinical* practice, for example, through staff training and modification of staff behaviours, but also through changing *organisational practice* by focusing on aspects such as; leadership, use of data to inform practice, de-briefing, consumer involvement, communication, staff learning approaches and staff wellbeing. In this context, differences were evident between trauma informed approaches utilised in that approaches such as The Six Core Stategies© (Huckshorn et al., 2005) or Trauma Systems Therapy (Saxe et al., 2007) incorporate an ‘in-built’ organisational change component, which were designed to support the implementation of a clinical component, while other training strategies utilised did not. Irrespective of this, eight papers reported on the use of some form of implementation strategy, such as Hale and Wendler (2020) use of The Iowa Model for Evidence Based Practice (Buckwalter, 2017). These ‘whole systems’ approaches were utilised in each study to varying degrees but identifying a causal relationship between the separate components of these approaches was not an objective of the included studies. Only one study reported on any attempt to measure the fidelity to an intervention (Boel-Studt, 2017) and closely replicating any of the approaches used in the included papers would be challenging due to a generalised approach to describing the interventions and due to the variety of contexts in which these interventions were used. As well as methodological differences, there was also variance in how the outcomes were measured and reported in terms of timelines and/or in type of restraints. Timelines for examining reductions in seclusion and restraint where reported, either retrospectively or prospectively, ranged from six months (Azeem et al., 2017) to ten years (Azeem et al., 2015). Where applicable, results were either reported as a percentage, or numerical reduction in restrictive practices. In all papers except two, it was reported that there were reductions in the use of restrictive practices following the implementation of a trauma informed approach.

Table 3 provides a summary of the intervention type and outcomes in each of the included studies. Detail on specific training content for each intervention was not provided in any of the studies but was described as being underpinned by a range of different approaches. Four of the included studies (Azeem et al., 2015, 2017; Caldwell et al., 2014; Hale & Wendler, 2020) utilised The Six Core Strategies© (Huckshorn et al., 2005) approach. In all of these studies, significant reductions in the use of seclusion and physical or mechanical restraint were reported. This included a 100% reduction for the use of mechanical restraint (Azeem et al., 2015) and reductions for physical restraint or seclusion ranging from 41% (Hale & Wendler, 2020) to 88% (Azeem et al., 2015) over various time periods. Two studies reported using trauma-informed training content as a component of their intervention (Denison et al., 2018; Marrow et al., 2012). Denison et al. (2018) reported significant improvements in staff attitudes to seclusion and restraint following training (p = 0.047), while Marrow et al. (2012) recorded a five-fold decrease in the use of restraint in a service-wide intervention group versus a control group. The approach utilised by Marrow and colleagues (2012) was aimed at enhancing environmental, staff and service-user strategies to reduce the use of restraint.

**Table 3:** Summary Table for Intervention Type and Outcome[s]

Author	Intervention Type	System-wide intervention [s]	Replication of educational content possible	Fidelity to Intervention Strategy was measured	Outcome
Azeem et al. (2017)	The Six Core Strategies© (Huckshorn et al., 2005)	Yes	No	No	Reduced seclusion/restraint from 93 episodes six months pre-intervention to 31 episodes six months post intervention
Azeem et al. (2015)	The Six Core Strategies© (Huckshorn et al., 2005)	Yes	No	No	Over a ten-year period; Decreased use in mechanical restraints by 100% and 0% in final 3 years. Physical restraints were reduced by 88%
Boel-Studt (2017)	Traditional Treatment v’s Trauma-Informed Treatment	Yes [for both interventions]	No	Yes	Over a 9-month period, the trauma informed cohort had higher incidents of restraint but lower incidents of seclusion when compared to the traditional group
Brown et al. (2013)	Trauma Systems Therapy (Saxe et al., 2007)	Yes	No	No	It was reported that: One site reduced seclusion, restraint and injury episodes from 80 to under 10 in the first 7 months. A second site reduced in rates of restraint from a peak of 42.98 per 1000- patient days to 7.59, over an 11-month period
Caldwell et al. (2014)	The Six Core Strategies© (Huckshorn et al., 2005)	Yes	No	No	Over an 8 yr period, it was reported that site 1 reduced the use of mechanical restraints by 100%, physical restraints by 87%, and seclusion by 67%. Over a 2-year period, site 2 reduced restraints per month from 49 to 0
Denison et al. (2018)	1-h tailor-made educational programme for staff	No	No	No	Reported statistically significant change in attitudes towards seclusion and restraint following training
Hildago et al. (2016)	2 ½ DAYS PATHS training- underpinned by Trauma Systems Therapy (Saxe et al., 2007) and Playfullness training (Life is good Inc., 2012)	Yes	No	No	Across 3 sites; from baseline measurement to 12 months statistically significant reductions in the number of restraints was reported
Marrow et al. (2012)	Treatment as usual v’s 1-day training & 2-day Trauma-informed TARGET (Ford & Russo, 2006) training for staff followed by 3 months supervision and follow up. Service-users were also provided with TARGET training	Yes	No	No	At T1, both TAU and TARGET cohorts used restraint at the same rate. Over a 12-month period post intervention the TAU group used restraint at a rate 5 times greater than the TARGET group
Hale and Wendler (2020)	One-day educational general trauma training and two-day training on Trauma-effect regulation; Intervention based on The Six Core Strategies© (Huckshorn et al., 2005)	Yes	No	No	At 6 months post intervention there was a 41% reduction in the use of restraint and seclusions. At 12 months post intervention, this had increased to a 50% reduction from pre-intervention

Two studies reported the on the use of Trauma Systems Therapy (Brown et al., 2013; Hidalgo et al., 2016) one of which, had a play-based focus (Hidalgo et al., 2016). Both of these studies reported reductions in restraint and seclusion over periods ranging from 7 months (Brown et al., 2013) to 12 months (p = 0.359) (Hidalgo et al., 2016). Finally, one quasi-experimental study which implemented two ‘system-wide’ interventions trauma-based CBT and what was described as ‘traditional treatment’ (Boel-Studt, 2017) found that the trauma informed group had higher incidents of restraint (p < 0.01) but lower incidents of seclusion (p < 0.001) when compared to the control group. The rationale for the increases of restraint in the trauma informed group could not be fully explained by the authors, and it was suggested that this could be attributed to sample characteristics. In this study, other outcomes, such as levels of functioning, were significantly improved in the trauma informed group.

**Fig. 2:**
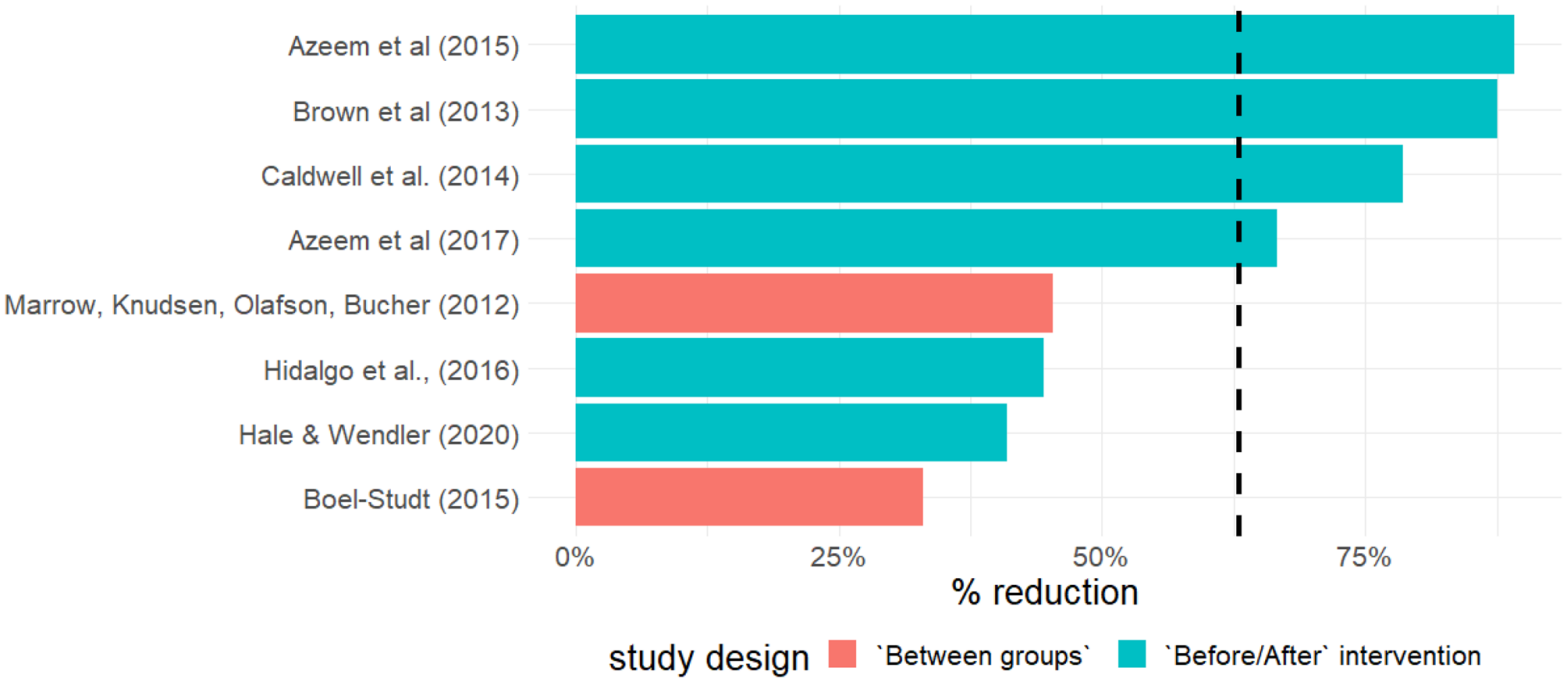
Percentage reduction reported in violent/aggressive events

Despite the heterogeneity of the included studies and the inability to complete a meta-analysis due to the lack of a common effect size estimate, in 8 out of the 9 included studies, the percentage reduction in observed aggressive/violent events is illustrated in Fig. 2. For this, data on numbers of all reported aggressive/violent events regardless of their type were assessed. Where studies used a repeated measure design (Azeem et al., 2015, 2017; Brown et al., 2013; Caldwell et al., 2014; Hale & Wendler, 2020; Hidalgo et al., 2016) the number of events at the beginning (before intervention) and end of the reporting period (after intervention) was used to calculate the percentage reduction. For studies with an between groups design (Boel-Studt, 2017; Marrow et al., 2012) percent reduction was calculated based on the number of events in the experimental versus control group.

The mean reduction in reported aggressive/violent events across all studies is 60,72%. The highest reduction is observed in the publication of Azeem et al. (2015), in which combined mechanical and physical restrain events were reduced by 89,2% after 10-year post-intervention period. Similarly, after transformation of beta coefficient of Zero-Inflated Poisson regression predicting restraint and seclusion Incidents by Boel-Studt (2017) 89% event reduction is observed, associated with the intervention compared to the control group. The lowest estimated value observed is based on the data of Hale and Wendler (2020) who reported a percentage reduction of combined seclusion and restraint of 41%, 6 months after an intervention.

## Discussion

The aim of this systematic review was to synthesize the available evidence on the use of trauma-informed interventions in reducing coercive practices in child and adolescent residential settings. This review provides an overview and synthesis of the literature which was not previously available. Owing to the study designs, which were largely case study-based or quasi-experimental, it is not clear which aspect of the interventions or the implementation strategies, such as increased data reporting to staff, had the greatest impact on the reported outcomes. Furthermore, owing to a lack of randomization, it is difficult to draw definitive conclusions about causal associations between interventions/approaches and outcomes (Schweizer et al., 2016). This methodological weakness is in stark contrast with the extant evidence related to trauma-informed interventions in adult populations, a number of studies have adopted a randomized controlled design (Han et al., 2021). A much more robust evidence base is warranted to support the use of trauma-informed interventions with child and adolescent populations.

Only one study (Boel-Studt, 2017) measured fidelity to intervention strategy, while no study provided sufficient information on the educational content, making replication of the initiative/intervention challenging. Intervention fidelity – where consistent delivery ensures that the same information is provided to all participants (Bonar et al., 2020) – is central to ongoing quality improvement. If fidelity to intervention is not measured, it is not possible to accurately assess the quality of a study or quality improvement initiative (Connelly, 2019). Future trauma-informed initiatives/interventions need to consider the standardization of content and delivery prior to implementation, in addition to transparent reporting of processes.

In this study we found that TIC based interventions mostly focused on changing the *clinical* or *organizational* practices, such as, staff behaviour or leadership. One way to bolster the impact of trauma-informed approaches may be to adopt a whole-systems strategy, evident in all included papers in the current review. However, it should be noted that there were inconsistencies in how studies employed whole systems strategies, and it is not possible to assess the complex relationship between organisational culture and the introduction of trauma-informed approaches. Chelagat et al. (2019) reported that there is often limited return on investment in training as a consequence of low application of knowledge gained. To effect real and sustained positive change, rather than (and prior to) the introduction of training programmes, organisational deficits need to be addressed, and the allocation of requisite resources warrants careful deliberation (Azeem et al., 2017; Kelly et al., 2017). Future studies focussed on children or adolescents need to be more strategic when embedding trauma-informed approaches within the wider organisational structure with the aim of fostering a whole-systems level commitment. Moreover, rigorous methodological approaches should be adopted to measure the multifaceted uptake of these approaches to evaluate where and how trauma-informed approaches can have the greatest impact. Bryson et al. (2017) discus several implementation challenges and developed a program theory of trauma informed practice implementation that includes the aspects of leadership, staff support, inclusion of patients and families, outcome orientation and alignment of policy and practice.

This review has identified the potential benefit of utilising a trauma informed approach when caring for children and adolescents who exhibit challenging behaviour during inpatient care provision. Evidence suggests that children and adolescents who exhibit behaviours that challenge have experienced a high incidence of childhood and intergenerational trauma (Ivanov et al., 2011). Coercive approaches have been described as ‘retraumatising’ for young people with a history of adversity (SAHMSA, 2014) and can seriously challenge the therapeutic process health outcomes and future help seeking behaviour (Bloom, 2013). The multifaceted nature of implementing trauma informed care has challenged the capacity of research studies in this review to conclusively identify that using this approach reduced coercive practices in this cohort, with further study indicated. However, children and adolescents who require inpatient care are likely experiencing significant health challenges that require them to be cared for away from their families and carers, thus represent a vulnerable group. It is therefore imperative that robust methodological approaches are developed and utilised to identify ways that children and adolescents can be cared for in ways that not only ensures their physical, but also their emotional safety, while in receipt of care. Mental Health care in child and adolescent inpatient units should always strive to be as respectful and empowering as possible, maintaining a safe and trustful environment, while respecting the child’s integrity. This implies keeping interventions that have the power to leave patients feeling shameful, angry, or victimized to a minimum (Perers et al., 2021).

### Limitations

This review is not without limitations. A ‘hierarchy of effectiveness’ for trauma informed approaches or training cannot be determined due to heterogeneity of study designs, lack of information relating to sampling and settings, and variance in the interventions used and reporting of results. Arguably, measurement of the effectiveness of more widely utilised Trauma informed strategies, such as The Six Core Strategies © (Huckshorn et al., 2005) is also methodologically challenging due to the wide range of variables, all of which will have influenced results across various organisational settings (Lewis et al., 2019). Moreover, excluding studies conducted among youths over the age of 19 years and not searching the grey literature and trial registries could have led to study selection bias. The small number of studies included in this review *also* limits the generalisability of the results to other practice settings.

## Conclusion

Results from the included studies suggest that the use of a trauma-informed approach underpinned by an organisational change or implementation strategy have the potential to positively impact on reducing coercive practices from staff, who work with young people that present with behaviours that challenge. Information innervations are likely to be associated with reduction of restriction practices. Further robust research, using implementation science, that has strong theoretical underpinnings is needed to further determine the impact of trauma informed interventions on the use of seclusion, restraint and coercive practice with children and adolescents. Utilisation of a humanistic approach such as TIC to address challenging behaviour, can potentially transform some of the most difficult aspects of care provision and subsequently positively impact young service users experience of care and improve staffs experience of care provision.
